# Supplementary material for: Gene expression alterations in testicular biopsies from males with spermatogenesis arrest identified by transcriptome analysis
Source: PLoS One. 2025 Sep 12;20(9):e0332025. doi: 10.1371/journal.pone.0332025 (PMC12431239; doi:10.1371/journal.pone.0332025)
Supplement: S1 Table — Control samples with OA, n = 9, Spermatogenic arrest (SA), n = 22, Mean values were reported with their corresponding standard deviation of the mean (SD), Statistical significance was determined using a non-parametric Mann–Whitney U test, with a significance threshold set at P < 0.05. (DOCX) [file pone.0332025.s001.docx]

**Supplementary S1 Table |** Clinical Characteristics of Included Subjects

| Parameters | OA | SA | P-value |
| --- | --- | --- | --- |
| Age (years) | 39.5 ± 3.2 | 35.9 ± 4.7 | 0.0913 |
| Testosterone (ng/dL) | 953 ± 115.3 | 431 ± 201.4 | <0.0001 |
| Luteinizing hormone (uIU/mL) | 7.32 ± 0.56 | 10.08 ± 7.47 | 0.3811 |
| Follicle-stimulating hormone (mIU/mL) | 11.17 ± 0.75 | 18.08 ± 14.99 | 0.2747 |
| Prolactin (ng/mL) | 20 ± 1.25 | 8.66 ± 2.98 | <0.0001 |
| Creatinine (mg/dL) | 1.99 ± 2.9 | 0.7013 ± 0.28 | 0.036 |
| Glucose serum (mg/dL) | 100.2 ± 8.7 | 91.93 ± 23.28 | 0.4055 |
| Sodium (mEq/L) | 116.8 ± 56.7 | 132.7 ± 11.55 | 0.2028 |
| Potassium (mEq/L) | 4.222 ± 0.53 | 3.623 ± 1.261 | 0.2707 |
| Bilirubin (mg/dL) | 1.06 ± 0.1705 | 0.648 ± 0.372 | 0.0144 |

- Control samples with OA, n = 9, Spermatogenic arrest (SA), n = 22
- Mean values were reported with their corresponding standard deviation of the mean (SD).
- Statistical significance was determined using a non-parametric Mann–Whitney U test, with a significance threshold set at P < 0.05.
