# Supplementary material for: Gene expression alterations in testicular biopsies from males with spermatogenesis arrest identified by transcriptome analysis
Source: PLoS One. 2025 Sep 12;20(9):e0332025. doi: 10.1371/journal.pone.0332025 (PMC12431239; doi:10.1371/journal.pone.0332025)
Supplement: S2 Table — provides comprehensive details regarding the control samples used in the comparison with our SA samples, including chromosomal analysis results, and diagnosis. (DOCX) [file pone.0332025.s002.docx]

**Supplementary S2 Table |** Details of Control Samples from Published Datasets of Obstructive Azoospermic Males Following Vasectomy Reversal

| Sample ID | Reason for TESE Procedure | Diagnosis | Age  at Biopsy | Karyotype | RNA Sequencing Datasets |
| --- | --- | --- | --- | --- | --- |
| FC1 | Vasectomy Reversal | Vasectomy | 52 | 46, XY | GSM6041956 |
| FC2 | Vasectomy Reversal | Vasectomy | 49 | 46, XY | GSM6041957 |
| FC3 | Vasectomy Reversal | Vasectomy | 50 | 46, XY | GSM6041958 |
| FC4 | Vasectomy Reversal | Vasectomy | 39 | 46, XY | GSM6041959 |
| FC5 | Vasectomy Reversal | Vasectomy | 37 | 46, XY | GSM6041960 |

S2 table provides comprehensive details regarding the control samples used in the comparison with our SA samples, including chromosomal analysis results, and diagnosis.
