# Supplementary material for: Gene expression alterations in testicular biopsies from males with spermatogenesis arrest identified by transcriptome analysis
Source: PLoS One. 2025 Sep 12;20(9):e0332025. doi: 10.1371/journal.pone.0332025 (PMC12431239; doi:10.1371/journal.pone.0332025)
Supplement: S3 Table — represents the BioProject accession number corresponding to our samples, together with the accession numbers of each sample. (DOCX) [file pone.0332025.s003.docx]

**Supplementary S3 Table |** Accession number of RNA-seq data deposited in the BioProject database.

| Sample ID | BioProject accession  number | Sample accession  number |
| --- | --- | --- |
| FC1 | SPRJNA825978 | SAMN27546557 |
| FC2 |  | SAMN27546556 |
| FC3 |  | SAMN27546555 |
| FC4 |  | SAMN27546554 |
| FC5 |  | SAMN27546553 |
| SA1 | PRJNA1233842 | SAMN47280701 |
| SA2 |  | SAMN47280702 |
| SA3 |  | SAMN47280703 |
| SA4 |  | SAMN47280704 |
| SA5 |  | SAMN47280705 |

S3 table represents the BioProject accession number corresponding to our samples, together with the accession numbers of each sample.
