# Supplementary material for: Gene expression alterations in testicular biopsies from males with spermatogenesis arrest identified by transcriptome analysis
Source: PLoS One. 2025 Sep 12;20(9):e0332025. doi: 10.1371/journal.pone.0332025 (PMC12431239; doi:10.1371/journal.pone.0332025)
Supplement: S4 Table — shows the qPCR primers used to assess the expression levels of each gene. These primers were designed in the exon-exon junctions to inhibit binding to DNA sequences. (DOCX) [file pone.0332025.s004.docx]

**Supplementary S4 Table |** qPCR primer sequences and annealing temperatures

| Gene ID | Forward Primer Sequence | Reverse Primer Sequence | Annealing Temp. |
| --- | --- | --- | --- |
| FOS | GCAGGACTTCTGCACGGAC | CTGGAGATAACTGTTCCACCTTG | 60°C |
| FOSB | ACTCCAGGCGGAGACAGATC | GAAGCCATCTTCCTTAGCCG | 60°C |
| RGS1 | GATGCTGCTAAACAAATCAATATTG | GCGCTTGATACTATCTGTTAATTCC | 60°C |
| CXCL8 | GTTTTTGAAGAGGGCTGAGAATTC | GTATGTTCTGGATATTTCATGGTACAATG | 55°C |
| TNP2 | CTCAGCTCCATAGCAACTCTCAG | GTGGGAGTTCATAGTCTTTTTGTG | 55°C |
| LINC02314 | GTAAAATGGAACTGAATGAGCGG | GGGAATTAGATGTGTTTGTGCTC | 55°C |
| SPRR2C | CAACAGTAGCAATGTAAGCAGCCC | GGTGTCACCAGAGGATATTTCTGC | 55°C |
| C16orf78 | GCTCCCGAGAAGCAAAAGC | GTCTGTATCAGATTTCTTGGGGC | 55°C |
| GAPDH | GTCAACGGATTTGGTCGTATTG | GGAACATGTAAACCATGTAGTTGAGG | 55°C |

S4 table shows the qPCR primers used to assess the expression levels of each gene. These primers were designed in the exon-exon junctions to prevent amplifying DNA regions.
