# Supplementary material for: Gene expression alterations in testicular biopsies from males with spermatogenesis arrest identified by transcriptome analysis
Source: PLoS One. 2025 Sep 12;20(9):e0332025. doi: 10.1371/journal.pone.0332025 (PMC12431239; doi:10.1371/journal.pone.0332025)
Supplement: Attachment — Submitted filename: Reviewer_Comments_PONE_D_25_14380.docx [file pone.0332025.s005.docx]

# Reviewer Comments – Manuscript PONE-D-25-14380

Title: Transcriptome analysis reveals altered gene expression in testicular biopsies from males with spermatogenesis arrest

The manuscript presents a well-designed and technically sound transcriptomic study investigating differential gene expression in testicular biopsies from patients with non-obstructive azoospermia (NOA) and spermatogenesis arrest (SA). The authors performed RNA-seq and RT-qPCR validation to identify key genes potentially implicated in spermatogenesis impairment. The findings are relevant to the field of reproductive biology and male infertility, and the results are generally well presented and supported by appropriate statistical analyses.

## Strengths:

- The study is ethically approved and based on well-defined patient groups.

- The combination of RNA-seq and RT-qPCR strengthens the reliability of the findings.

- Functional and pathway enrichment analyses are adequately performed.

- The discussion is comprehensive and supported by relevant literature.

## Minor Revisions Recommended:

1. Figure Quality and Titles:

- All figures appear blurry and difficult to interpret in the current version. Please provide high-resolution images.
 - Figures lack titles. Each figure should include a clear title and legend, following the journal’s formatting guidelines.

2. Figure Captions and Labels:

- Ensure all axes, labels, and color scales in figures are clearly visible and annotated. For example, in the volcano and heatmap plots (Figure 2), gene names and significance levels should be readable.

3. English Language and Style:

- The manuscript is written in acceptable English, but minor language polishing is recommended to enhance clarity and flow.
 - Consider revising long paragraphs into shorter, more concise sections.

4. Clarify PCA Results:

- In the Results section (lines 281–282), double-check the PCA variance explanation percentages. PC1 is usually the largest component, and the statement “PC1 at 8.3% and PC2 at 87.3%” may need correction or clarification.

5. Supplementary Tables:

- Make sure all supplementary tables (S1–S4) are clearly referenced in the main text and accessible to readers.

6. Discussion Enhancements:

- The discussion around CXCL8 and RGS1 could be expanded to include more specific references about their role in germ cell biology or testicular inflammation if available.

## Conclusion:

I recommend minor revision. The manuscript provides meaningful insights into the molecular underpinnings of spermatogenic arrest and male infertility. Once the figures are improved, and minor clarifications are made, the manuscript would be suitable for publication in PLOS ONE.
