# Supplementary material for: Gene expression alterations in testicular biopsies from males with spermatogenesis arrest identified by transcriptome analysis
Source: PLoS One. 2025 Sep 12;20(9):e0332025. doi: 10.1371/journal.pone.0332025 (PMC12431239; doi:10.1371/journal.pone.0332025)
Supplement: Attachment — Submitted filename: Response file.docx [file pone.0332025.s006.docx]

**Dear Editorial Team and Reviewers**

**We sincerely thank the editorial team and reviewers for their thoughtful evaluation of our manuscript and for the valuable comments and suggestions. We greatly appreciate the time and effort invested in reviewing our work. The constructive feedback has significantly contributed to improving the clarity, scientific rigor, and overall quality of the manuscript. In response, we have thoroughly addressed each comment and made the corresponding revisions, as detailed in the point-by-point response. We trust that the updated version meets your expectations and strengthens the impact of our study within the field.**

**Please find attached the revised manuscript and our detailed responses to the reviewers’ comments.**

**Sincerely,**

**Dr. Osamah Batiha**
Associate Professor of Cell and Molecular Genetics
Jordan University of Science and Technology, Irbid, Jordan
Email: oybatiha@just.edu.jo | Tel: +962-2-7201000 ext. 26818

**Editor Comments:**

When submitting your revision, we need you to address these additional requirements.

1. Please ensure that your manuscript meets PLOS ONE's style requirements, including those for file naming.

We apologize for the earlier oversight in formatting. The revised manuscript now fully adheres to PLOS ONE’s style requirements, including file naming conventions, as outlined in the provided templates.

2. Thank you for stating the following financial disclosure: “Deanship of Research at Jordan University of Science and Technology (Grant No. 2022044).” Please state what role the funders took in the study.  If the funders had no role, please state: "The funders had no role in study design, data collection and analysis, decision to publish, or preparation of the manuscript." If this statement is not correct you must amend it as needed. Please include this amended Role of Funder statement in your cover letter; we will change the online submission form on your behalf.

The Deanship of Research at Jordan University of Science and Technology provided financial support for this project (Grant No. 2022044). However, the funders had no role in study design, data collection and analysis, decision to publish, or preparation of the manuscript. This statement has been included in the cover letter as requested.

3. Your ethics statement should only appear in the Methods section of your manuscript. If your ethics statement is written in any section besides the Methods, please move it to the Methods section and delete it from any other section. Please ensure that your ethics statement is included in your manuscript, as the ethics statement entered into the online submission form will not be published alongside your manuscript.

The ethics statement has been retained solely within the Materials and Methods section under “Sample Collection,” and has been removed from all other sections of the manuscript, in accordance with the journal’s guidelines.

4. Please include captions for your Supporting Information files at the end of your manuscript, and update any in-text citations to match accordingly. Please see our Supporting Information guidelines for more information: http://journals.plos.org/plosone/s/supporting-information.

Captions for all Supporting Information files have been added to the end of the manuscript, and in-text citations have been updated accordingly to ensure consistency with PLOS ONE’s guidelines.

**Reviewer #1:**

**Summary of the Study**

- The authors conducted a comparative transcriptomic study to identify differentially expressed genes in non-obstructive azoospermic men with spermatogenesis arrest, compared to obstructive azoospermic (OA) men with normal spermatogenesis.
- They performed RNA sequencing on testicular tissue biopsies (n=5 SA, n=5 OA) and validated selected DEGs via RT-qPCR in an expanded sample cohort (n=22 SA, n=9 OA).
- They found 1,915 DEGs (337 upregulated, 1,578 downregulated), identifying eight key genes (FOS, TNP2, CXCL8) potentially involved in SA.
- Functional enrichment (GO and KEGG) analyses highlighted pathways such as MAPK signaling and p53 signaling as critical in spermatogenesis regulation.

**Merits**

Novelty and Relevance

- The study targets a significant clinical problem—male infertility due to spermatogenic arrest.
- By focusing on NOA with testicular histological staging, the authors address an underexplored but critical area.

Methodological Rigor

- RNA-seq quality control, alignment (STAR), and statistical analysis (DESeq2, Benjamini-Hochberg) were correctly implemented.
- External public datasets were used as OA controls due to RNA quality issues, which is a practical solution given sample constraints.
- RT-qPCR validation across a broader sample base strengthens the findings.

Comprehensive Bioinformatics Analysis

- Functional annotations (GO/KEGG), PCA, volcano plots, and correlation matrices provide strong visual and statistical support for the findings.

Data Accessibility

- Raw sequencing data has been deposited in the SRA (BioProject PRJNA1233842), fulfilling PLOS ONE’s open data policies.

**Limitations and Areas for Improvement**

**A. Major Concerns**

1. **Small Sample Size for RNA-Seq (n=5 SA): This is a serious limitation for robust differential expression analysis. Statistical power is restricted, potentially inflating false positives/negatives.**

We appreciate the reviewer’s observation regarding the small sample size used for RNA-seq and fully acknowledge that this is a limitation of our study. We recognize that a limited sample number reduces statistical power and may increase the risk of false positives or negatives. However, several unavoidable constraints impacted our ability to include more samples during the study period:

- Informed consent was not obtained from all patients undergoing testicular biopsy, which restricted sample availability.
- Histopathological examination revealed that fewer than 30% of the collected biopsies met the inclusion criteria for spermatogenesis arrest.
- Biopsy samples with observable sperm were preserved for IVF use and thus were not available for research purposes.
- Additionally, a number of RNA and cDNA samples failed to meet the quality control thresholds necessary for RNA-seq analysis.

Given these challenges, we strategically utilized RNA-seq as a **discovery platform** to identify candidate genes of interest rather than to draw definitive conclusions from the sequencing alone. To enhance the robustness of our findings, we performed **RT-qPCR validation in an expanded, independent cohort**, which included a significantly larger number of samples. This validation step was critical in confirming the differential expression of the selected genes. This two-phase approach careful candidate identification through RNA-seq followed by broader validation via RT-qPCR serves as a methodologically practical solution given the sample size constraints. The consistency observed between the RNA-seq and RT-qPCR results helps to mitigate the impact of the limited initial sample size and supports the reliability of the key findings.

2. **Control Sample Source Inconsistency: The OA samples used for RNA-seq were from a public dataset, while qPCR controls were locally collected. This raises concerns of ethnic variability, and technical heterogeneity despite efforts to align sequencing protocols. If authors do not have to do with this, then they should consider it as major limitation of their study.**

We recognize this as a major limitation and have acknowledged it in the Discussion section, and we have now clearly stated this issue in the Limitations section as well. To clarify further: samples with observable sperm during biopsy were preserved for IVF, including the majority of fine-needle aspiration (FNA) samples with normal sperm counts. However, we managed to collect five FNA control samples after obtaining patient consent. Unfortunately, due to the small tissue volume, we encountered technical issues during RNA extraction and cDNA synthesis. The RNA yield was insufficient for RNA sequencing. As a result, we were unable to use local samples for RNA-seq controls and resorted to using publicly available datasets.

To minimize variability, we carefully selected a public dataset that most closely matched our own sample characteristics, including clinical features and inclusion criteria. While we acknowledge the potential for ethnic and technical heterogeneity, we used the RNA-seq data primarily as a discovery tool to identify potential candidate genes, rather than to draw definitive conclusions. To strengthen the validity and robustness of our findings, we conducted RT-qPCR validation in an expanded and independent local cohort with a substantially larger sample size. This validation step was critical in confirming the differential expression of selected genes and reinforcing the biological relevance of our results.

3. **Gene Selection Justification: The basis for selecting the eight genes (4 upregulated, 4 downregulated) is not fully justified biologically or statistically. Were these the top DEGs by p-value, fold-change, or literature relevance? A more systematic, data-driven method, like using adjusted p-value alongside biological relevance, would be stronger. Authors should discuss why they have emphasized on these genes leaving other significantly differentially expressed genes.**

We sincerely thank the reviewer for this valuable comment. As widely recognized in the literature, the selection of genes for RT-qPCR validation is typically based on a combination of statistical significance, magnitude of differential expression (fold change), and biological relevance to the disease or condition of interest. In our analysis, we applied stringent criteria to ensure the robustness of our gene selection. Specifically, we included only genes with a **p-value < 0.05** and a **log2 fold change greater than +2 or less than –2**, thereby focusing on genes with both high statistical significance and substantial expression differences. From this filtered list, we further prioritized genes with documented or strongly suggested roles in spermatogenesis and testicular function, as supported by existing publications. The eight selected genes (four upregulated and four downregulated) fulfilled all of these criteria. Additionally, the number of genes selected for validation was partly influenced by experimental constraints, particularly the limited quantity of cDNA obtained from some clinical samples. While it was possible to validate a greater number of targets in samples with sufficient cDNA, this was not feasible in samples with lower yields, which limited the overall number of genes we could consistently analyze across the entire cohort. We have now clarified this selection strategy in the revised manuscript, and we hope this addresses the reviewer’s concern.

4. **While RT-qPCR validation on 17 more SA samples is good, the lack of stratification by arrest stage in this cohort is a weakness. Genes might behave differently and express differently across these stages. If authors could not do to rectify this, then at least they should consider it as their limitation.**

We thank the reviewer for this thoughtful comment. We fully agree that gene expression may vary across different stages of spermatogenic arrest, and we recognize that the lack of stratification in our RT-qPCR validation cohort represents a limitation of the study. This has now been clearly acknowledged in the revised manuscript. To address this issue, we performed differential expression analysis from the RNA-seq data using two approaches:

- First, by comparing the full SA group (n = 5) to the OA control group (n = 5).
- Second, by analyzing each arrest stage separately specifically, four samples with spermatid arrest and one with spermatogonia arrest against the control group and against each other. Although this analysis revealed stage-specific DEGs, the small sample size in each subgroup limited the statistical power and prevented robust conclusions. **As such, these findings were not included in the manuscript but can be provided upon request.**

While our RT-qPCR validation was based on the overall SA vs. OA comparison, we also examined gene expression across the three SA subgroups for the eight validated genes. This analysis did not show statistically significant differences among the subgroups. **Supporting data for this analysis are available and can be shared upon request.** We hope this clarification is helpful, and we have now explicitly noted the absence of stage-based stratification in the RT-qPCR cohort as a limitation in the revised manuscript.

**B. Minor Concerns**

1. **Figures are not clear, may be due to submission-related deformation. Figure and Table legends should be more descriptive to allow understanding without the main text.**

Thank you for pointing this out. We have improved the resolution and overall quality of all figures to ensure clarity in the revised manuscript. Additionally, we have revised the figure and table legends to be more descriptive and self-explanatory, enabling readers to understand the content without referring to the main text.

2. **The term “spermatogenesis arrest” is sometimes abbreviated as SA and sometimes written out consistency is advised.**

We appreciate this observation. The manuscript has been carefully reviewed and revised for consistency, and the abbreviation “SA” is now used uniformly after its first definition.

**Suggestions for Improvement**

3. **Clarify statistical thresholds and gene selection: Mention precise thresholds for DEG selection (adjusted p-value, log2FC) and how the final eight genes were shortlisted.**

Thank you for this valuable suggestion. In our analysis, we applied clear filtering criteria: genes with p-values ≥ 0.05 and log2 fold change between -2 and +2 were excluded. We then ranked the remaining genes by descending log2FC. From this list, we selected genes with both high fold changes and p-values < 0.05. These genes were also evaluated for biological relevance to spermatogenesis based on existing literature. The final number of genes selected for RT-qPCR validation (eight) was determined in part by technical limitations, particularly the available cDNA quantity, which allowed us to perform reliable duplicate reactions across all samples.

4. **Discuss broader implications: Expand on how these candidate genes could be targeted or screened clinically, or how they relate to known pathways in spermatogenic regulation.**

We appreciate this suggestion. In response, we have added a paragraph in the Discussion section highlighting the broader implications of our findings. This includes the potential of the validated genes to serve as biomarkers for diagnosis, as well as their relevance to pathways involved in spermatogenic regulation. We also briefly comment on how these targets may be further explored for therapeutic interventions in future studies.

5. **Line no 92 to 100: This part may not be required and seems to be irrelevant with the article. This portion can be omitted.**

Thank you for this helpful observation. We agree that the content in lines 92 to 100 was not directly relevant to the core objectives of the manuscript. We have removed this section and replaced it with more pertinent information.

6. **Materials and Methods: Inclusion criteria regarding case sample selection should be more precise. Whether case patients were checked for Y chromosome microdeletion or not is to be confirmed. Y chromosome microdeletion is an important consideration as it can strongly influence the gene expression profile.**

Thank you for raising this important point. We have now clarified the inclusion and exclusion criteria in the **Materials and Methods** section. We confirm that patients included in the study were not screened for Y chromosome microdeletions. We acknowledge that this is a limitation of the study and have noted it accordingly in the revised manuscript.

7. **Quality of the figures should be enhanced.**

We appreciate this comment. The quality and resolution of all figures have been enhanced in the revised submission to improve visual clarity and readability.

**Reviewer #2:**

The manuscript presents a well-executed and promising study on spermatogenesis arrest using transcriptomics. However, above-mentioned issues are to be and must be addressed. This is a technically sound and well-structured manuscript investigating the transcriptomic landscape of spermatogenesis arrest (SA) in non-obstructive azoospermia (NOA) patients. The use of high-throughput RNA-sequencing and validation by RT-qPCR on testicular tissue samples represents a valuable approach to better understand molecular mechanisms underlying male infertility. The authors clearly define their objectives, and the methods are described in sufficient detail to allow reproducibility. The integration of bioinformatics analysis, including differential gene expression, GO and KEGG pathway enrichment, and PCA, is appropriate and strengthens the conclusions drawn. The results are consistent and well presented. The correlation between RNA-seq and RT-qPCR data is particularly convincing. **However, I have a few minor revisions to suggest:**

We thank **Reviewer #2** for the thoughtful and encouraging feedback. We appreciate your recognition of the strengths in our study and have carefully addressed each of your comments as outlined below

1. **Figures:** All figures currently lack descriptive titles or clear legends. Please provide informative captions for each figure, explaining what is shown, group comparisons, statistical indicators, and the meaning of colors or symbols used.
   The quality of the figures is suboptimal. They appear blurry, which makes them difficult to interpret. Please upload high-resolution versions.

We thank the reviewer for this observation. In the revised manuscript, all figure legends have been rewritten to be more descriptive and self-contained, clearly explaining the comparisons, statistical indicators, and visual elements used. Additionally, we have replaced the original figures with high-resolution versions to ensure clarity and readability.

2. **Supplementary Material**: Ensure that all supplementary tables referenced in the text (e.g., Table S1, S2, S3, S4) are available and properly labeled in the submission package.

Thank you for noting this. The supplementary tables have now been properly labeled and included in the revised submission package, with consistent references throughout the manuscript.

3. **Clarify the use of control data**: Since RNA-seq control samples were obtained from previously published datasets, please clearly state any differences in data acquisition or batch effects that may have occurred and how they were addressed in the analysis.

We appreciate the reviewer’s attention to this important detail. The control samples used for RNA-seq comparison were obtained from a previously published dataset that utilized the same sequencing platform (Illumina NovaSeq6000 System, paired-end sequencing) as our samples. Moreover, the control samples were histologically confirmed to have normal spermatogenesis. To minimize technical variability, we ensured that library preparation protocols and sequencing parameters were closely matched. We have clarified this point in the revised Materials and Methods section.

4. **Gene validation rationale**: While the selection of top up/down-regulated genes is valid, a brief explanation for choosing these specific genes for RT-qPCR (e.g., biological relevance, novelty, expression intensity) would enhance clarity.

Thank you for raising this point. We selected genes for RT-qPCR validation using a combination of statistical and biological criteria. Genes with p-values ≥ 0.05 and log2 fold change between –2 and +2 were excluded. The remaining DEGs were ranked by descending log2FC, and those with the highest expression changes and p-values < 0.05 were shortlisted. In addition, we prioritized genes with known or suggested roles in spermatogenesis and male reproductive biology, based on prior literature. The final number of genes selected (four upregulated and four downregulated) was also guided by experimental constraints, particularly the quantity of available cDNA. All qPCR reactions were performed in duplicate to ensure accuracy and reproducibility. We have clarified this rationale in the revised Results section.

5. **English Language and Formatting**: The manuscript is generally well written. However, a minor revision by a native English speaker or language editor would help improve fluency and consistency. For example, correct terms like “SPRR2C, a pseudogene” → “a small proline-rich protein-2C, a pseudogene product involved in...”, or revise redundant phrases for conciseness.

We are grateful for this suggestion. The manuscript has been carefully reviewed and edited by a native English speaker to improve fluency, grammar, and clarity throughout. Specific language corrections, including those suggested (e.g., gene annotations and concise phrasing), have been implemented in the revised version.

6. **Discussion**: The discussion is comprehensive and rich in references. However, a brief paragraph summarizing the clinical implications of the findings (e.g., potential diagnostic or therapeutic targets) at the end would strengthen the conclusion.

We thank the reviewer for this thoughtful suggestion. We have added a new concluding paragraph to the **Discussion** section summarizing the potential clinical relevance of our findings: “Importantly, differentially expressed genes (DEGs), such as *TNP2, FOS, FOSB, CXCL8, and RGS1*, could serve as diagnostic biomarkers or therapeutic targets for male infertility in cases of spermatogenesis arrest. *TNP2* downregulation could serve as an indicator of impaired chromatin remodelling, while elevated *CXCL8* levels might indicate oxidative stress-induced infertility. As MAPK and GPCR pathways are dysregulated, manipulating them directly could offer novel treatment strategies. These findings not only contribute to an understanding of the molecular basis of SA but also aid in creating clinically useful interventions aimed at improving diagnosis and patient outcomes in NOA.”

7. **Overall Comment**: Overall, this is a solid and promising manuscript that contributes new insights to the understanding of testicular transcriptomics in male infertility. I recommend minor revision before acceptance.

We sincerely thank the reviewer for this encouraging assessment. We have addressed all suggestions and revised the manuscript accordingly. We hope the changes meet the reviewer’s expectations and further strengthen the quality of the work.
